# Evidence-based decision making and promotion of physical activity among directors of local health departments

**DOI:** 10.11606/S1518-8787.2018052000379

**Published:** 2018-11-14

**Authors:** Leonardo Augusto Becker, Cassiano Ricardo Rech, Adriano Akira Ferreira Hino, Rodrigo Siqueira Reis

**Affiliations:** I Pontifícia Universidade Católica do Paraná. Grupo de Pesquisa em Atividade Física e Qualidade de Vida. Curitiba, PR, Brasil; II Universidade Federal do Paraná. Programa de Pós-Graduação em Educação Física. Curitiba, PR, Brasil; III Universidade Federal de Santa Catarina. Centro de Desportos. Departamento de Educação Física. Florianópolis, SC, Brasil; IV Pontifícia Universidade Católica do Paraná. Programa de Pós-Graduação em Tecnologia em Saúde. Curitiba, PR, Brasil; V Universidade Federal Tecnológica Federal do Paraná. Programa de Pós-Graduação em Educação Física. Curitiba, PR, Brasil; VI Washington University in St. Louis. Brown School. Prevention Research Center. St Louis, MO, USA; VII Pontifícia Universidade Católica do Paraná. Programa de Pós-Graduação em Gestão Urbana. Curitiba, PR, Brasil

**Keywords:** Decision Making, Formulation of Policies, Health Plans and Programs, Health Promotion, Exercise and Movement Techniques, Unified Health System, Tomada de Decisões, Formulação de Políticas, Planos e Programas de Saúde, Promoção da Saúde, Técnicas de Exercício e de Movimento, Sistema Único de Saúde

## Abstract

**OBJECTIVE:**

To describe the steps involved in evidence-based decision making for the implementation of programs aimed at the promotion of physical activity.

**METHODS:**

It is a descriptive, cross-sectional study with quali-quantitative approach, held with municipal health secretaries chosen deliberately by regional health representatives of the state of Paraná. A total of 27 secretaries participated in a telephone interview consisting of 17 open questions. Content analysis was conducted according to the categories of an evidence-based decision-making model consisting of seven steps.

**RESULTS:**

None of the participants employed every step of the evidence-based decision-making model. The steps that were most often mentioned included: evaluation of the program (33.3%), use of evidence from the literature (22.2%) and identification of the problem (22.2%). The steps that were reported the least included: quantification of the problem (14.8%), development and prioritization of actions (14.8%), development of the plan of action (14.8%) and evaluation of the community (3.7%).

**CONCLUSIONS:**

The use of evidence-based decision making in the context of the promotion of physical activity was shown to be incipient among the health secretaries of the state of Paraná. We suggest widening dissemination and training on the use of evidence-based decision making among municipal administrators to increase the effectiveness of actions for promotion of physical activity.

## RESUMO

**OBJETIVO:**

Descrever o emprego das etapas da tomada de decisões baseada em evidências para implementação de programas de promoção da atividade física.

**MÉTODOS:**

Trata-se de um estudo descritivo, transversal, com abordagem quali-quantitativa, realizado com secretários municipais de saúde escolhidos intencionalmente por representantes das regionais de saúde do estado do Paraná. Ao todo, 27 secretários participaram de uma entrevista telefônica composta por 17 questões abertas. A análise de conteúdo foi conduzida segundo as categorias de um modelo de tomada de decisões baseada em evidências composto de sete etapas *.*

**RESULTADOS:**

Nenhum dos participantes empregou todas as etapas do modelo de tomada de decisões baseada em evidências. As etapas mais mencionadas foram: avaliação do programa (33,3%), uso de evidências da literatura (22,2%) e identificação do problema (22,2%). As etapas menos reportadas foram: quantificação do problema (14,8%), desenvolvimento e priorização de ações (14,8%), desenvolvimento do plano de ação (14,8%) e avaliação da comunidade (3,7%).

**CONCLUSÕES:**

O emprego da tomada de decisões baseada em evidências no contexto da promoção da atividade física apresentou-se incipiente entre os secretários de saúde do estado do Paraná. Sugere-se ampliar a disseminação e o treinamento para o uso de tomada de decisões baseada em evidências entre os gestores municipais a fim de ampliar a efetividade das ações de promoção da atividade física.

## INTRODUCTION

Despite the relative consistency in the evidence on interventions for promotion of physical activity (PA) ^1^ ^,^ ^2^ , their implementation and maintenance on a large scale is still a challenge in many countries, particularly those with low and medium income ^1^ ^,^ ^2^ . In general, the lack of political support and adaptation of operations to the sociocultural context has been considered as one of the most challenging aspects ^1^ . These aspects can be even more complex for the promotion of PA alongside the Brazilian public health system, since administrators are faced with high demand of priorities and have little time available for actions in health ^3^ .

The use of instruments for decision making in the context of health promotion can improve both the daily routine of administrators as well as the access to health services. Municipal administrators can understand more easily the demands and the needs of local communities and are therefore central people in the development of local actions for the promotion of health ^4^ ^,^ ^5^ . The permanent contact between administrators, health committees and technical teams is important because it allows health decisions to be based on scientific evidence and on the local reality, and not only on the political context inherent to their role ^6^ . Evidence-based decision making (EBDM) has been considered important and effective ^7^ and is defined as the “use of the best current scientific evidence for making decisions about the care of communities and populations in the context of protection, improvement, maintenance of health and prevention of diseases” (Brownson et al. ^8^ , p.3).

The use of EBDM is recent in the field of PA and occurs predominantly in high-income countries ^7^ . In these countries, the use of EBDM is more common among health managers with higher education, who work in large cities and receive resources, training and support for this purpose ^7^ ^,^ ^8^ . In Brazil, the use of EBDM in programs for promotion of PA is still incipient ^8^ and even the instruments employed in this process, such as the Logic Model ^4^ or evaluation models such as RE-AIM ^5^ , are not used routinely in the management of these programs. In Brazil, the instruments have been applied in already-implemented programs, limiting their initial planning ^9^ ^,^ ^10^ . In addition, such tools do not explore in detail the decision-making process that involves the implementation and evaluation of the programs ^11^ . An evidence-based public health model consisting of seven steps has been adopted in an attempt to analyze EBDM among administrators ^12^ ^,^ ^13^ . Understanding the use of EBDM in the Brazilian context, particularly considering the complexities of PA programs in the country – such as inadequate funding, the almost absence of PA surveillance systems in small and medium-sized municipalities and lack of qualified teams for the promotion of PA ^14^ –, represents an important step for the implementation and maintenance of these programs. Thus, the primary objective of this study was to describe the employment of the EBDM steps for the implementation of programs to promote physical activity in municipalities of the state of Paraná. As a secondary objective, we compared the application of the decision-making steps between municipalities with positive experiences and municipalities with difficulties in the implementation of programs for promotion of physical activity.

## METHODS

### Study Design

This study is part of the project Local practices and use of evidence in the prevention of chronic non-communicable diseases in the state of Paraná. It is a descriptive, cross-sectional study with quali-quantitative approach, held with municipal health secretaries of the state of Paraná. The study was approved by the Ethics Committee for Research with Human Beings of Pontifícia Universidade Católica do Paraná (Process 130,240).

### Population and Sample

The state of Paraná is located in the Southern region of Brazil, has 11,219,013 inhabitants distributed in 399 municipalities and occupies a land area corresponding to 199,880 km ^2^ . It is the fourth largest economy in the country and its human development index is 0.749, considered high for the Brazilian context ^15^ . In terms of health, the municipalities of Paraná are administratively organized in 22 regions, which are responsible for the state’s health administration. The regional administration is performed by a health representative and a supporter, who are responsible for identifying the population’s health priorities, carrying out planning and managing the municipalities’ health system in partnership with the municipal health secretariats (MHS). Each municipality has a health secretariat managed by a secretary.

The selection of participants took place in two stages. In the first, the representatives of the state’s 22 regional administrations were contacted by telephone, since they are privileged actors in the localities for having access to the reality of each municipality, as well as to the actions that are carried out in them. Two representatives did not accept participating in or did not attend the interviews. The 20 representatives contacted indicated, according to the perception and knowledge of their location, the municipalities that had positive experiences and those that showed difficulties in the implementation of programs for the promotion of PA. In all, 33 municipalities were indicated, 17 with positive experiences and 16 with difficulties. In the second stage, the indicated MHS were invited to participate in the study. Among the 33 MHS invited, six (four municipalities with positive experiences and two with difficulties) (18.2%) declined. Thus, the final sample was composed by 27 MHS: thirteen municipalities that had positive experiences and 14 that had difficulties in the implementation of actions for promotion of PA.

### Data Collection

The data were collected through telephone interviews between the months of July and August 2015. The secretaries’ contact information (name, telephone number, email address, telephone for messages and municipality) was made available by the Council of Municipal Health Secretaries of Paraná (COSEMS-PR) ^6^ . Initially, an email was sent to the secretaries, with information about the study and an invitation to participate in it. Then, each MHS was contacted by phone to clarify possible doubts about the participation, and an interview was scheduled according to the availability of each participant.

The telephone interviews had an average 30-minute duration (minimum = 20 minutes; maximum = 45 minutes) and consisted of 17 open questions, organized into seven blocks: i) socio-demographic information; ii) health priorities in regional health administration; iii) knowledge of evidence-based interventions for prevention of chronic non-communicable diseases (NCD); iv) barriers to evidence-based decision making; v) evidence-based support for NCD prevention; vi) steps of evidence-based decision making for NCD prevention and vii) steps of evidence-based decision making for promotion of PA. The questionnaire was developed jointly by one international and three national researchers with experience and publications in the field of health, NCD and public management, and aimed to standardize the information pertaining to the decision-making process.

The transcription of the interviews was carried out by two auxiliary researchers, having been verified by the study’s coordinator. The secretaries’ anonymity was maintained, because the data that could identify them were replaced by codes (S1, S2, S3, etc.)

### Study Variables and Data Analysis

The data were analyzed using a descriptive approach and by carrying out content analysis ^16^ . Participants answered the following question: “Could you please describe the process of planning, development and implementation of programs for promotion of physical activity in your municipality?”. Answers were categorized according to the seven EBDM steps defined *a priori* , according to the model proposed by Brownson ^8^ , described below:

- Step 1 – Evaluating the community: identification of health priorities and comparison with the country’s surveillance data (e.g., System of Surveillance and Risk Factors and Protection Against Non-Communicable Chronic Diseases via Telephone Investigation ^17^ ).
- Step 2 – Identifying the problem: characterization via intersectoral meetings with the administrators to verify the program’s implementation needs.
- Step 3 – Quantifying the problem: qualification of the target population (e.g., age, gender, race, physical activity level, co-morbidities).
- Step 4 – Seeking evidence in the literature: mapping of scientific evidence about PA programs in scientific journals and databases (PubMed, SciELO, Virtual Health Library), government documents and websites ^18^ .
- Step 5 – Developing and prioritizing actions: identification of the need for political support for the program’s implementation, as well as of the cost for implementation, maintenance and definition of the necessary structure.
- Step 6 – Developing a plan of action: use of a logical model for the program’s implementation ^4^ . At this stage, the administrators must identify the goals (short, medium and long term), as well as the types of activities carried out, the work team, the forms of intervention, the structure and the materials needed.
- Step 7 – Evaluating the program: identification of the program’s dissemination, adherence by the population and results in accordance with the proposed objectives (both in relation to the population’s health needs as well as to financial aspects).

## RESULTS

In all, 27 MHS of the state of Paraná participated in this study, 13 from municipalities with positive experiences and 14 from municipalities with difficulties in the implementation and execution of programs for promotion of PA. The participants were predominantly women (56.6%), between 41 and 59 years old (55.5%), with training in the field of health (48.1%), having occupied the position for a period between 13 and 36 months (51.8%) and acting in medium-sized municipalities (55.5%), with between 10,001 and 50,000 inhabitants ( Table ).

**Table:** Profile of the municipal health secretaries of municipalities with positive experiences and difficulties in the promotion of physical activity. Paraná, Brazil, 2015. (n = 27)

Variable	Categories	n	%
Gender	Male	12	44.4
	Female	15	55.6
Age group	20–40	9	33.3
	41–60	15	55.5
	≥ 60	3	11.2
Academic background	Humanities	5	18.5
	Exact and Earth Sciences	4	14.8
	Biological and Health Sciences	13	48.1
	Engineering and technology	1	3.8
	No academic background	4	14.8
Time of professional performance	Up to 12 months	7	29.2
	13–36 months	14	51.8
	≥ 37 months	6	19.0
Population size	< 10,000 inhabitants	8	29.7
	10,001–50,000 inhabitants	15	55.5
	≥ 50,001 inhabitants	4	14.8
EBDM steps	Evaluating the community	1	3.7
	Identifying the problem	6	22.2
	Quantifying the problem	4	14.8
	Evidence in the literature	6	22.2
	Developing and prioritizing actions	4	14.8
	Developing a plan of action	4	14.8
	Evaluating the program	9	33.3
Number of EBDM steps reported by the secretaries			
	0	8	29.6
	1	9	33.4
	2	5	18.5
	3	5	18.5
	4 or more	0	0.0

EBDM: Evidence-based decision-making

In relation to the process of adoption of the EBDM steps for implementation of PA promotion programs, no secretary reported the application of all seven steps, and one in three secretaries did not apply any of the steps suggested by the literature. The steps most often employed by the secretaries were: evaluating the program (33.3%), using evidence from the literature (22.2%) and identifying the problem (22.2%). The steps that were applied the least in the municipalities investigated included: quantifying the problem (14.8%), developing and prioritizing actions (14.8%), developing a plan of action (14.8%) and evaluating the community (3.7%) ( Table ).

The MHS of municipalities with positive experiences mentioned the use of more EBDM steps than those that acted in municipalities with difficulties in the implementation of actions for promotion of PA ( Figure ). However, even among the MHS of municipalities with positive experiences, it was observed that less than one in four MHS used the steps: evaluating the community, identifying the problem, seeking evidence in the literature, developing and prioritizing actions and developing a plan of action. In general, the MHS of these municipalities used the steps: quantifying the problem and evaluating the program. Among the MHS of municipalities with difficulties in the promotion of PA, on the other hand, only four of the seven EBDM steps were mentioned: identifying the problem, seeking evidence in the literature, developing and prioritizing actions and evaluating the program ( Figure ).

**Figure:**
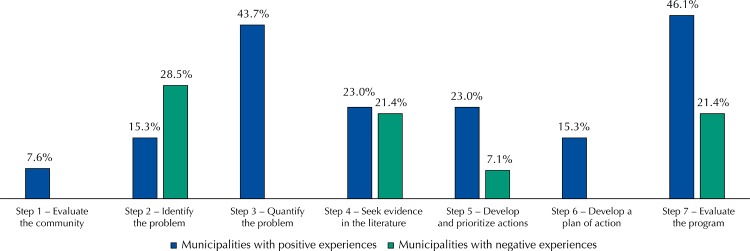
Steps for evidence-based decision making reported by municipal health secretaries of municipalities with positive experiences or with difficulties in the promotion of physical activity. Paraná, Brazil, 2015. (n = 27)

Box 1 and 2 show examples of reports of managers related to the application of the EBDM steps for promotion of physical activity.

**Box 1:** Reports of municipal health secretaries with positive experiences that highlight the process of planning, development and implementation of physical activity programs. Paraná, Brazil, 2015. (n = 13)

Steps	Reports
Step 1 – Evaluating the community	“The identification of the problem is very simple, and all programs face this physical activity issue. We verified a large number of obese people in the municipality. A survey has been carried out to find out the percentage of the overweight portion of the population {indicates the physical activity program}” S12
Step 2 – Identifying the problem	“The program was based on the course on the needs of the municipality” S1
Step 3 – Identifying the problem	“In relation to physical activity, in physical activity days, we have realized that there is greater adherence of the population” S7
Step 4 – Evidence in the Literature	“I think that if they {professionals who are present daily} propose something, it will be based on some sort of literary concept that has been written by someone, and that has yielded results. But in truth, we started to suggest the implementation of some actions and adapted them according to the demand of our municipality” S7
Step 5 – Developing and prioritizing actions	“The professionals who work there have to devise the planning, get to know the target audience, verify what is the method that will be developed and then hold monthly meetings to discuss if the planning is being followed” S10
Step 6 – Developing a plan of action	“Nowadays, we have the health academy coordinated by the physical education professional with support from the Family Health Support Center ( *Núcleo de Apoio à Saúde da Família* – NASF). So, the activities we carry out there include Pilates, dance lessons, lectures, counselling and activities developed with older adults, in addition to the care of and guidance to pregnant women. Planning and counselling are carried out in groups according to patient and age” S8
Step 7 – Evaluation of the programs	“On Friday afternoons we work with evaluation. So we identify a project, a program or more, and evaluate the situation. We are getting good results” S12

**Box 2:** Reports of municipal health secretaries with difficulties that highlight the process of planning, development and implementation of physical activity programs. Paraná, Brazil, 2015. (n = 14)

Step	Report
Step 2 – Identifying the problem	“A project was made. A survey based on the age group of the municipality’s population was conducted and it was observed that there are many people over 50 years old. So, the work developed at the family health care center would have to be adapted accordingly” S17
Step 4 – Evidence in the Literature	“We need to gather information in order to find out how it works, where it worked, and how it was able to achieve a good result” S15
Step 5 – Developing and prioritizing actions	“We have a health academy which was completed. So, professionals will be hired to work there. It is a federal government resource that was provided to the municipality. Now, we will define the professionals who will work there, as many professionals may be hired. By following the logic of developing the care of people with this health problem, we will define the professionals to be hired” S15
Step 7 – Evaluating the program	“Each one of them {patients} has a chart with stratification of risk for physical activity. It is not just any person who goes there, mainly older adults. So, we need to pay attention to this, and this evaluation is held monthly and quarterly” S20

## DISCUSSION

The study’s results indicate that none of the MHS interviewed employed all the EBDM steps, and that one in every three MHS did not apply any of the steps in the implementation of programs for promotion of PA. The implementation of the EBDM steps was more frequent among the MHS of municipalities with positive experiences compared to those with difficulties. The steps most often employed by the secretaries were: evaluating the program, using evidence from the literature and identifying the problem.

The results of this study indicate that the MHS have difficulty recognizing the local needs and including strategies to identify and quantify the problem, because these steps are fundamental for the application of EBDM ^8^ . These steps are followed to understand the community’s health priorities. It is possible, through data from the municipalities’ surveillance system, to establish the magnitude of the problem. Health priorities, the target population’s characteristics (age, sex, economic condition) and the organizations/sectors that are responsible for developing strategies to minimize the problem in question (e.g., physical inactivity) may be established. Although most municipalities have surveillance data regarding the most prevalent morbidities, such as: arterial hypertension, diabetes mellitus, obesity, among others, information about the practice of PA in small and medium-sized municipalities is still scarce ^19^ . Thus, it is clear that these steps are still little explored in the process of implementation of the PA programs, as mentioned in the following report:

“The identification of the problem is very simple, and all programs face this physical activity issue. We verified a large number of obese people in the population. A survey has been carried out to find out the percentage of the overweight portion of the population {indicates the physical activity program}” S12.

The main need indicated in the report was the fight against obesity, the practice of PA being recognized as an important aspect to reverse this situation, which is supported by the literature ^20^ . On the other hand, considering that PA is an important factor of protection against the main NCD ^21^ , its recognition as priority would be expected. However, the lack of a system for surveillance of PA levels in small and medium-sized cities makes the recognition of the physical inactivity issue’s magnitude difficult for administrators. Currently, only state capitals and the Federal District ^17^ , in addition to an expanded, although still restricted group of municipalities ^22^ ^,^ ^23^ , regularly monitor the adult population’s PA levels.

Another important result indicates a low rate of use of scientific evidence among the secretaries. The low rate of application of this step can be associated with little familiarity with the scientific language, difficulties of access to scientific evidence and lack of ability to develop a scientific evidence-based program ^24^ . In addition, the reports show that the actions for promotion of PA require the use of scientific evidence both by the administration and by the technical team: “each professional develops his/her action and seeks his/her own information!” S6. However, the rate of evidence use is still low and not institutionalized in the Municipal Health Secretariats. Seeing as the MHS are players in the municipalities’ health policies, it is essential that they use the best scientific evidence available to make decisions. Given this need, the Ministry of Health created the Evidence-Based Health Portal, that allows access to major scientific journals. Furthermore, a closer relationship between scholars and professionals in the field of health management is necessary so technical and scientific knowledge may be implemented in the formulation of PA programs. Interventions in the school environment, intersectoral actions, information campaigns and changes in the planning of the cities are concrete and contribute to the increase in the population’s PA levels ^2^ ^,^ ^25^ . In this way, the MHS could involve specialists in the field of development and implementation of PA programs. In addition, it is important to understand and evaluate the programs that already exist in the municipalities because, in many cases, the scientific evidence is based on day-to-day experiences (practice-based evidence) ^1^ . This fact is corroborated by a statement about the importance of understanding the best practices employed in the municipalities, because in case of success, they can be adapted to other locations: “But in truth, we started to suggest the implementation of some actions and adapted them according to the demand of our municipality” S7.

Indeed, it is important to understand how the programs are implemented (practice-based evidence), because they have high external validity. The results of this study show that the secretaries have difficulties in the following steps: developing and prioritizing actions and developing a plan of action, this being the most frequent among the MHS with difficulties in the promotion of PA, according to the reports presented.

“We discussed a couple of months ago that, during the Hiperdia meetings, it would be requested that nurses went for walks with the people from the group. So, this was the first idea we had. Next we will carry out an evaluation and see what else fits this profile” S4.

The difficulty in planning causes interventions to have little chance of being effective or sustainable, because, as identified in this study, the MHS have difficulties in assessing and identifying the community’s problems, since these steps are fundamental for the whole process of development of the plan of action. The actions often do not have well-defined goals and do not meet the population’s needs. In addition, many programs do not have sufficient funding or a qualified technical team ^1^ ^,^ ^14^ . Thus, some instruments might assist the MHS in the implementation of PA programs. Among them, there is the use of the Logic Model ^4^ , which is performed in six steps: i) participation of the interested parties; ii) description and development of the program’s planning; iii) evaluation of the program; iv) assessment of trustworthiness of the information; v) data analysis and conclusions; vi) implementation of the program and recommendations for its continuity ^4^ ^,^ ^26^ . Another instrument that can be used is the RE-AIM ^5^ model, which stands for Reach (characteristic of the target population, number of participants); Efficacy (intervention’s impact on the target population); Adoption (factors that influence the practice of PA, number of organizations that have implemented the program); Implementation (program’s consistency, appropriate structures, forms of dissemination); Maintenance (evaluation of the program in the short, medium and long term) ^4^ ^,^ ^5^ . However, the use of such instruments demands the involvement of professionals in the area of PA promotion.

The step that was most often mentioned by the participants was: evaluating the program. This result corroborates that of another study which shows that the assessment of the impact of the interventions and economic benefits are the main factors referred to by decision-makers in relation to the PA programs’ implementation ^27^ . The quantitative data obtained in this study are reinforced by the MHS’s reports: “Right at the start their weight is measured and, at the group’s request, their development is evaluated weekly” S4.

Despite its importance, the evaluation reported by the participants refers only to the population’s health indicators, which corroborates the literature ^28^ . In this way, it would be important that the secretaries assessed the program’s implementation through its dissemination and the population’s adherence to it. A study conducted in the city of Curitiba, Brazil, identified that 75.8% of the respondents were aware of the PA programs offered by the municipal city hall, but only 0.8% participated in them ^29^ . Moreover, the program’s impact on the change in the participants’ lifestyle may be evaluated ^8^ .

Generally speaking, although the analysis of the present study focused on the EBDM steps, the secretaries have no understanding of the process as a whole. It is necessary to train the MHS and the technical team so they may adopt EBDM as a process that will improve planning, cost-effectiveness, adherence and health indicators for the community.

The results of this study provide important information about the process of planning, implementation and development of PA among the MHS of the state of Paraná. However, the sample was obtained through an indication of the regional coordinators and contrasted the counties with positive experiences and those with difficulties in the actions for promotion of PA. However, the comparison between municipalities with extremes of experience and success in the promotion of PA does not clarify all the complexity and diversity that exists in the state’s municipalities. Thus, we cannot generalize the results for municipalities with medium performance in PA promotion actions.

The findings are based on reports of the administrators of each secretariat; it is possible that some response bias is present, as they could overestimate their knowledge of the subject. Considering that the results indicate low knowledge about the EBDM process, this bias would not change the conclusion of the study. Furthermore, no technical coordinators of the programs were interviewed to expand the possibilities of analysis and interpretation of the data.

The results of the study indicate that the employment of the EBDM steps to implement the PA promotion programs in municipalities of the state of Paraná is infrequent and limited to a few steps only. In addition, municipalities with prominent actions for the promotion of PA use more EBDM steps than municipalities with difficulties in the promotion of PA. We suggest the expansion of the information about the EBDM process in Brazil, especially among municipal administrators of PA promotion programs, because the implementation of its steps is an important mechanism for the success of these initiatives in Brazilian municipalities. Promoting training courses, expanding the source of high-quality information and facilitating the exchange of experiences between municipalities with more experience in PA actions are strategies that can be tested in Brazil for greater use of the EBDM process in the promotion of PA.
